# 699 Psychometric Testing of a New Outcome Measure for Hand Burn Injuries

**DOI:** 10.1093/jbcr/iraf019.328

**Published:** 2025-04-01

**Authors:** Andrea Mc Kittrick, Louise Gustafsson, Tenelle Hodson

**Affiliations:** Royal Brisbane and Women’s Hospital; Griffith University; Griffith University

## Abstract

**Introduction:**

A co-designed hand outcome measure was developed using a Participatory Action Research framework, involving expert clinicians and individuals with hand burn injuries. The measure contains 18 activities designed to capture elements of movement, grip and pinch strength that are missed by existing assessments. The aim of this study was to establish the clinical utility, face, and content validity of the measure.

**Methods:**

Clinicians from several facilities working in burns and individuals who had experienced hand burns were sought to test the outcome measure. A copy of the outcome measure was sent to clinicians. Individuals attending a burns centre with deep dermal or full thickness hand burns were invited to assess the outcome measure. All participants competed a survey post testing the outcome measure to determine the three constructs of interest.

**Results:**

Eight clinicians and 20 individuals with hand burn injuries tested the outcome measure. There was 100% agreement from clinicians and 85% agreement from individuals for face validity. Clinicians rated 16 of the activities included as relevant, individuals rated all 18 activities as relevant. Clarity of activities >75% was high for both participant groups.

**Conclusions:**

Clinical utility was measured in terms of appropriateness, accessibility, practicability, and acceptability. Clinicians reported agreement of 87.5% for all components. 95% of individuals reported agreement for practicability and 100% agreement for acceptability.

**Applicability of Research to Practice:**

A co-designed activity-based outcome measure demonstrated utility, face and content validity indicating potential appropriateness for use in practice. Further validity and reliability testing is required before implementation into standard practice can occur.

**Funding for the Study:**

2022 Foundation Research Project Grant

